# *Barley Stripe Mosaic Virus* (BSMV) Induced MicroRNA Silencing in Common Wheat (*Triticum aestivum L*.)

**DOI:** 10.1371/journal.pone.0126621

**Published:** 2015-05-08

**Authors:** Jian Jiao, Yichun Wang, Jonathan Nimal Selvaraj, Fuguo Xing, Yang Liu

**Affiliations:** Institute of Agro-Products Processing Science and Technology, Chinese Academy of Agricultural Sciences/Key Laboratory of Agro-Products Processing, Ministry of Agriculture, Beijing, P.R. China; Sabanci University, TURKEY

## Abstract

MicroRNAs (miRNAs) play important roles in growth, development, and response to environmental changes in plants. Based on the whole-genome shotgun sequencing strategy, more and more wheat miRNAs have been annotated. Now, there is a need for an effective technology to analyse endogenous miRNAs function in wheat. We report here that the modified *barley stripe mosaic virus* (BSMV)-induced miRNAs silencing system can be utilized to silence miRNAs in wheat. BSMV-based miRNA silencing system is performed through BSMV-based expression of miRNA target mimics to suppress miR159a and miR3134a. The relative expression levels of mature miR159a and miR3134a decrease with increasing transcript levels of their target genes in wheat plants. In summary, the developed approach is effective in silencing endogenous miRNAs, thereby providing a powerful tool for biological function analyses of miRNA molecules in common wheat.

## Introduction

Gene silencing has been known as one of reverse-genetic methods to analyze functional genomics for which exhaustive mutant collections are unavailable. MicroRNAs (miRNAs) and short interfering RNAs (siRNAs) are two major classes of silencing RNAs in plants and animals [1–4]. Plant miRNAs are especially important in controlling plant development and responses to biotic or abiotic stress [5–9]. These regulatory RNAs recognize specific target sequences based on sequence complementarity, resulting in translational repression or site-specific cleavage [7,10,11]. To date, a lot of miRNAs have been identified from different plant species [12]. With an increasing effort in miRNAs discovery by high-throughput sequencing and the completion of wholegenome sequences of several plant species, there is a growing need for functional genomics study of miRNAs [13]. However, the functions of identified or predicted miRNAs are largely unknown.

Understanding the functions of miRNAs *in vivo* requires an effective technology to block their activity. Recently, several alternative approaches have been developed for functional analyses of miRNAs in plants, including miRNA target mimicry (MIM) [14,15], short tandem target mimic (STTM) [15,16], transcriptional gene silencing of miRNA gene promoters [17], and artificial miRNA directed silencing of miRNA precursors [18,19]. Among them, miRNA silencing technology using MIM and STTM structure have received more attention [15]. The MIM technology was first reported in *Arabidopsis*. Non-protein-coding RNA *INDUCED BY PHOSPHATE STARVATION1* (*IPS1*), partially basepairing to miR399 with a three-nucleotide mismatch at miR399 cleavage site, can sequester miR399 and arrest its cleavage activity to its target *PHOSPHATE2* mRNA. Therefore, *IPS1* functions as noncleavable target mimic of miR399 [14]. In addition, another newly developed STTM technology is also an effective method to block miRNA function. The STTM structure, consisting of two mimicking small RNA target sites, can lead to the degradation of targeted small RNAs by small RNA-degrading nucleases [16].

Plant viral vectors have been widely utilized for transient gene expression or silencing in plants [20–22]. In *Nicotiana benthamiana* (*N*. *benthamiana*), a *cabbage leaf curl virus*-based vector for the overexpression of miRNAs [19] and a *tobacco rattle virus*-based vector for miRNA inactivation have been reported [15]. Viral vector-induced silencing techniques provide timesaving procedure for generating transient transgenic plants by allowing characterization of phenotypes that might be lethal knockout in stable transgenic lines. Therefore, these technologies have the potential to become an attractive and quick approach to uncover miRNA functions in plants, especially in those difficult for genetic transformation [15], such as wheat crops. However, virus-based miRNA silencing technology has not been developed in wheat.

*Barley stripe mosaic virus* (BSMV) is a hordeivirus with a tripartite genome, composed of the α, β, and γ RNAs [23]. It has been emerged as a VIGS vector for cereals [24,25] and generates a robust silencing response. BSMV-mediated VIGS system has been extensively used to investigate several protein-coding genes in both barley and wheat [26–28]. Recently, a modified BSMV-induced VIGS protocol has been reported, in which BSMV-α, β, γ RNAs are initially cloned in a binary vector respectively, and a target gene cDNA fragment is inserted into downstream of BSMV-γb strand via Ligation Independent Cloning (LIC) method. After that restructured three clones (BSMV-α, β, γ) are transiently transformated *N*. *benthamiana* in a *agrobacterium*-mediated manner [29].

Wheat is one of the most widely cultivated and consumed food crops in the world, with heterologous hexaploid genome composed of A, B and D sub-genomes. The complexity of the genome brings enormous challenges for study of molecular biology and genomics in common wheat [30]. One current challenge in wheat crops research is still to reveal the functions of all genes including miRNAs in whole genome, which could ultimately facilitate the identification of genes for important agronomical traits and the linkage between gene functions and specific traits across different varieties [31]. Compared with other plants, stable genetic transformation of wheat requires a longer period and complex operations. However, transient virus-induced gene silencing assists in evaluating gene functions before cross species introgression or stable transformation and displays several advantages when constitutive loss of gene function through stable transformation causes embryonic lethality. Therefore, it is urgent to develop a convenient toolbox for the induction of miRNAs silencing, and for functional analyses of miRNAs and their target genes in common wheat.

Here, we report the efficient utilization of BSMV vector in wheat to silence endogenous miRNAs by expressing miRNA target mimics. MIM or STTM structure is linked downstream of the stop codon of BSMV-γb open reading frame. Together with intermediary of argoinocubated *N*. *benthamiana*, BSMV-induced miRNAs silencing vector is used to knockdown of miRNAs and to verify the corresponding target genes in wheat, accompanied by more favourable experiment operation and more economy.

## Results

### Integration of BSMV-γb vector and *AtIPS1*-based MIM or STTM sequences

**Fig 1:**
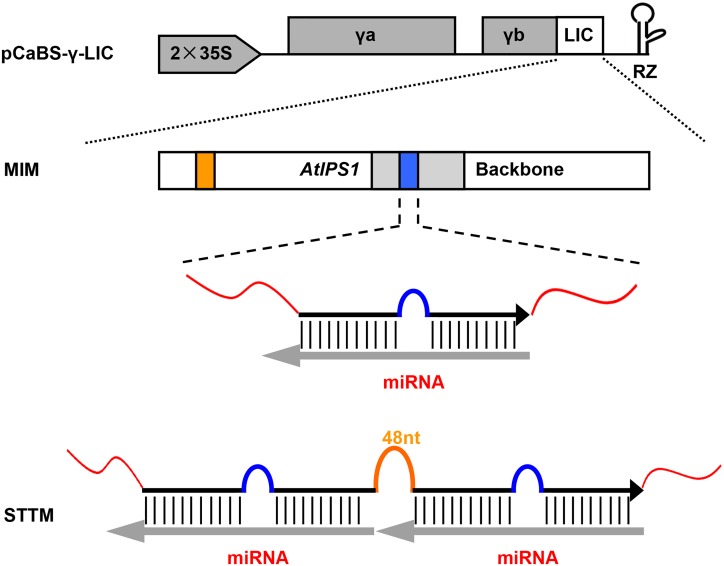
Schematic diagram of integrating BSMV-γb vector and *AtIPS1*-based MIM or STTM sequences. Modified BSMV-γb vector (pCaBS-γ-LIC) was shown in this figure. *AtIPS1*-based MIM or STTM sequences can be cloned into pCaBS-γ-LIC derivatives by the LIC reaction. MIM structure contained an *AtIPS1* backbone, but the target mimic motif of AthmiR399 was changed to that of corresponding miRNAs. STTM structure contained two tandem target mimics separated by a 48 nt imperfect stem-loop linker as described [16].

**Fig 2:**
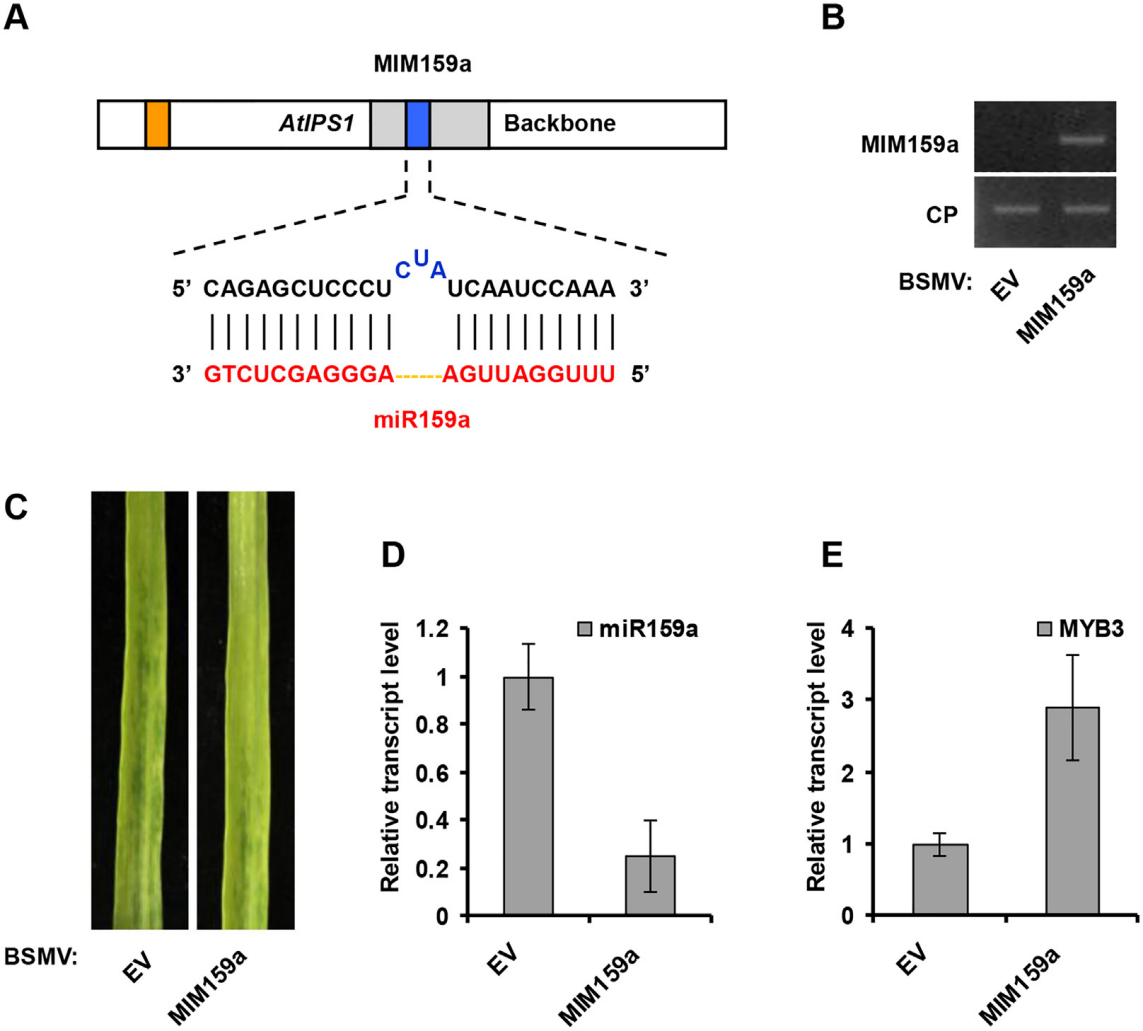
BSMV-based miR159a silencing using *AtISP1*-based miRNA target mimicry in wheat. (**A**) Diagrammatic representation of MIM159a structure. (**B**) Semiquantitative RT-PCR assays detection of MIM159a structure expression in wheat infected with BSMV-EV and with BSMV-MIM159a. CP, coat protein of BSMV. (**C**) The 4^th^ leaves of wheat infected with BSMV-EV (left) and with BSMV-MIM159a (right) were photographed at 20 dpi. (**D**) Stem-loop RT-PCR together with real-time quantitative PCR (qPCR) detection of mature miR159a relative transcript level in wheat infected with BSMV-EV and with BSMV-MIM159a. Error bars represented standard error (SE) of three representing experiments from four replicates. (**E**) Real-time RT-PCR analysis of mRNA levels of miR159a target *TaMYB3* in BSMV-EV control and plants expressing MIM159a structure. Error bars representing SE were calculated from three replicates.

**Fig 3:**
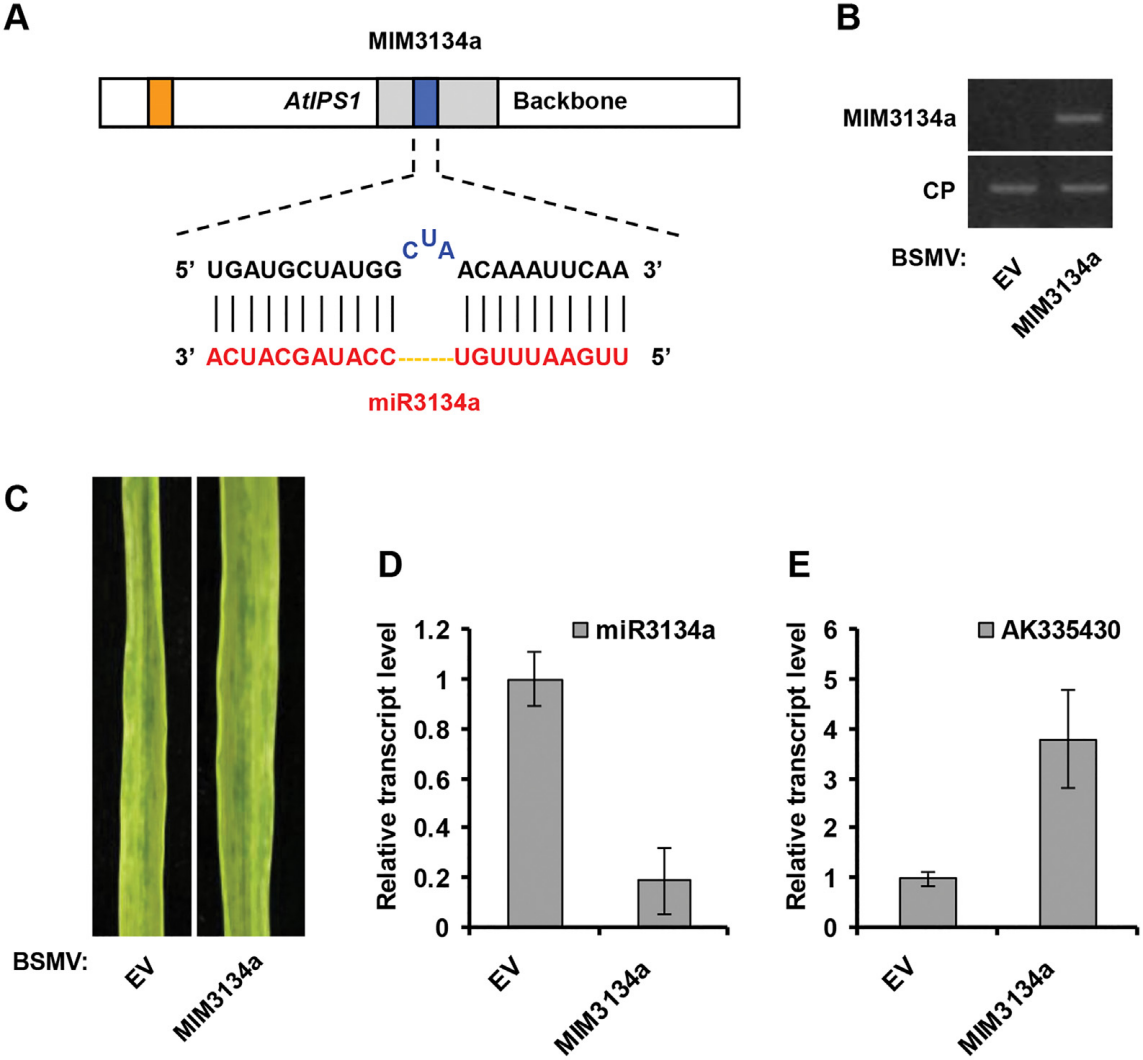
BSMV-based miR3134a silencing using *AtISP1*-based miRNA target mimicry in wheat. (**A**) Diagrammatic representation of MIM3134a structure. (**B**) Semiquantitative RT-PCR assays detection of MIM3134a structure expression in wheat infected with BSMV-EV and with BSMV-MIM3134a. CP, coat protein of BSMV. (**C**) The 4^th^ leaves of wheat infected with BSMV-EV (left) and with BSMV-MIM3134a (right) were photographed at 20 dpi. (**D**) Stem-loop RT-PCR together with real-time qPCR detection of mature miR3134a relative transcript level in wheat infected with BSMV-EV and with BSMV-MIM3134a. Error bars represented SE of three representing experiments from four replicates. (**E**) Real-time RT-PCR analysis of mRNA levels of miR3134a target AK335430 in BSMV-EV control and plants expressing MIM3134a structure. Error bars representing SE were calculated from three replicates.

Modified BSMV vectors (pCaBS-α, pCaBS-β, pCaBS-γ-LIC derivatives) were employed in this study [29]. *AtIPS1*-based MIM or STTM structure was linked downstream of the stop codon of BSMV-γb (pCaBS-γ-LIC) open reading frame via LIC method (Fig 1). BSMV-based miRNA silencing experiment was performed as described in Materials and Methods section. When two-leaf stage wheat plants were infected with BSMV harboring MIM or STTM sequences, MIM or STTM sequences were detected in upper new leaves at 14–21 days post inoculation (dpi) through semiquantitative reverse transcription (RT)-PCR analysis (Figs 2B and 3B). Therefore, this modified BSMV vector can be used to express RNAs such as MIM and STTM sequences as outlined in Fig 1.

### miR159 and miR3134 can be silenced by BSMV carrying MIM sequences

miR159 is conserved in monocot or dicot plants while miR3134 belongs to wheat- and barley-specific miRNAs family. To test whether BSMV carrying a miRNA target mimic (MIM) can suppress miRNA activity, we used the modified BSMV vector to express *AtIPS1*-based target mimic against miR159a (MIM159a, for silencing of miR159a using *AtIPS1* sequence as backbone) or against miR3134a (MIM3134a, for silencing of miR3134a using *AtIPS1* sequence as backbone), and then cloned it into pCaBS-γ-LIC vector to generate BSMV-MIM159a and BSMV-MIM3134a, respectively (Figs 2A and 3A). BSMV-based miRNA silencing experiment procedures can be found in Materials and Methods section. BSMV symptoms were visible in the upper noninoculated leaves after two-leaf stage wheat plants were infected with BSMV carrying MIM sequences (Figs 2C and 3C). Furthermore, semiquantitative RT-PCR assays indicated that MIM159a and MIM3134a sequences were expressed in BSMV-MIM159a and BSMV-MIM3134a infected plants, respectively (Figs 2B and 3B). Stem-loop RT-PCR together with real-time PCR assays showed a decline in the relative transcript level of mature miR159a and miR3134a in BSMV-MIM159a and BSMV-MIM3134a infected plants, respectively (Figs 2D and 3D). It is known that *TaMYB3* is one target of miR159a in wheat, and miR159a is highly abundant and regulates MYB transcription factors involved in plant development and disease resistance [32]. We also found that miR3134a could partially basepair a candidate target gene (AK335430, Genebank number) using NCBI nucleotide blast tool. Thus, real-time RT-PCR was employed to analyze the mRNA level of *TaMYB3* and AK335430. Indeed, the level of the *TaMYB3* and AK335430 mRNA were much higher in MIM159a- and MIM3134a-expressing plants than in controls infected with BSMV empty vector (BSMV-EV; Figs 2E and 3E). Therefore, BSMV vectors carrying MIM sequences were effective in the silencing of endogenous miRNAs in wheat.

### miR159 and miR3134 can be suppressed by BSMV expressing STTM sequences

**Fig 4:**
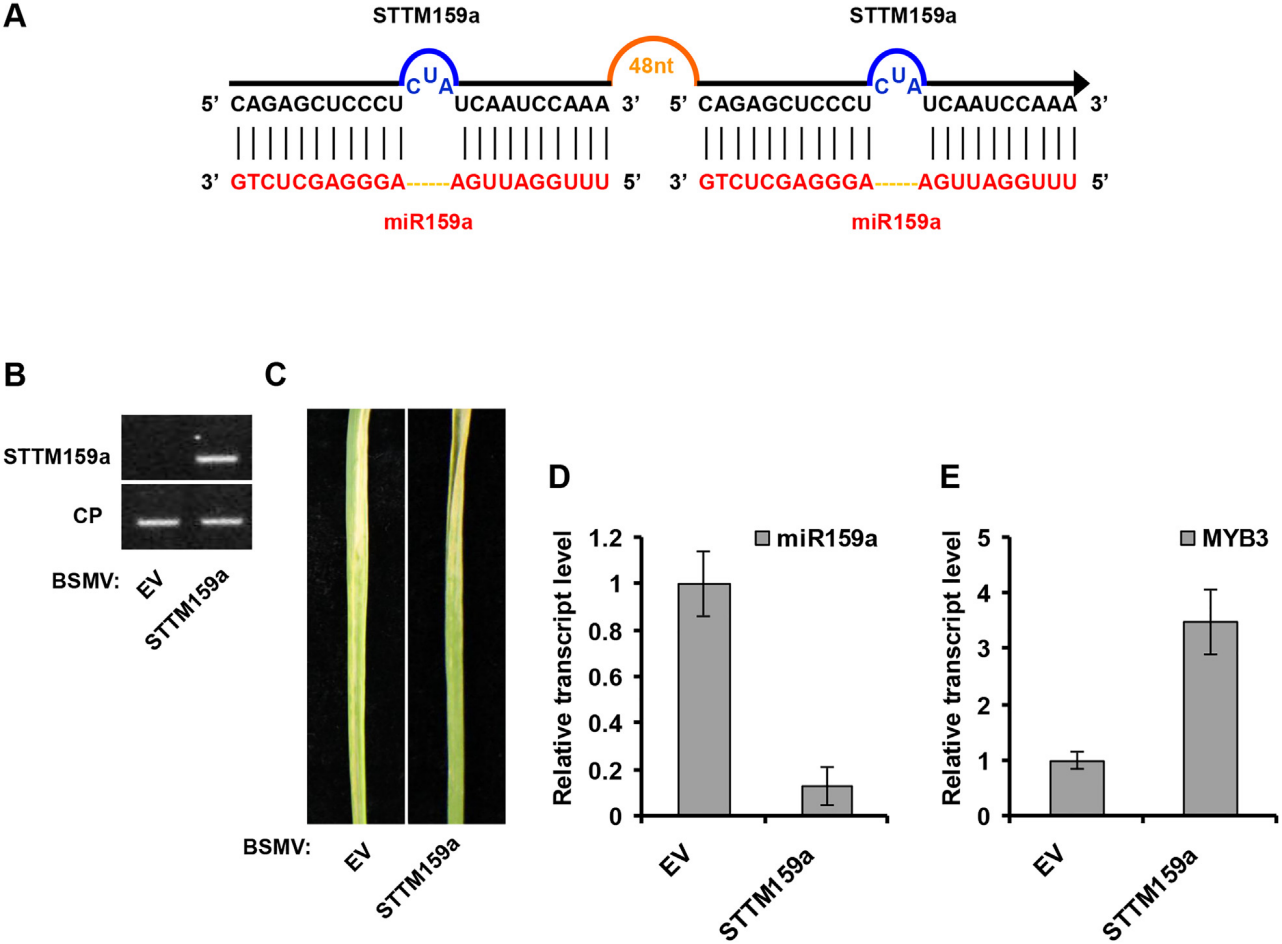
BSMV-based miR159a silencing using the STTM approach in wheat. (**A**) Diagrammatic representation of STTM159a structure. (**B**) Semiquantitative RT-PCR assays detection of STTM159a structure expression in wheat infected with BSMV-EV and with BSMV-STTM159a. CP, coat protein of BSMV. (**C**) The 4^th^ leaves of wheat infected with BSMV-EV (left) and with BSMV-STTM159a (right) were photographed at 20 dpi. (**D**) Stem-loop RT-PCR together with real-time qPCR detection of mature miR159a relative transcript level in wheat infected with BSMV-EV and with BSMV-STTM159a. Error bars represented SE of three representing experiments from four replicates. (**E**) Real-time RT-PCR analysis of mRNA levels of miR159a target *TaMYB3* in BSMV-EV control and plants expressing STTM159a structure. Error bars representing SE were calculated from three replicates.

**Fig 5:**
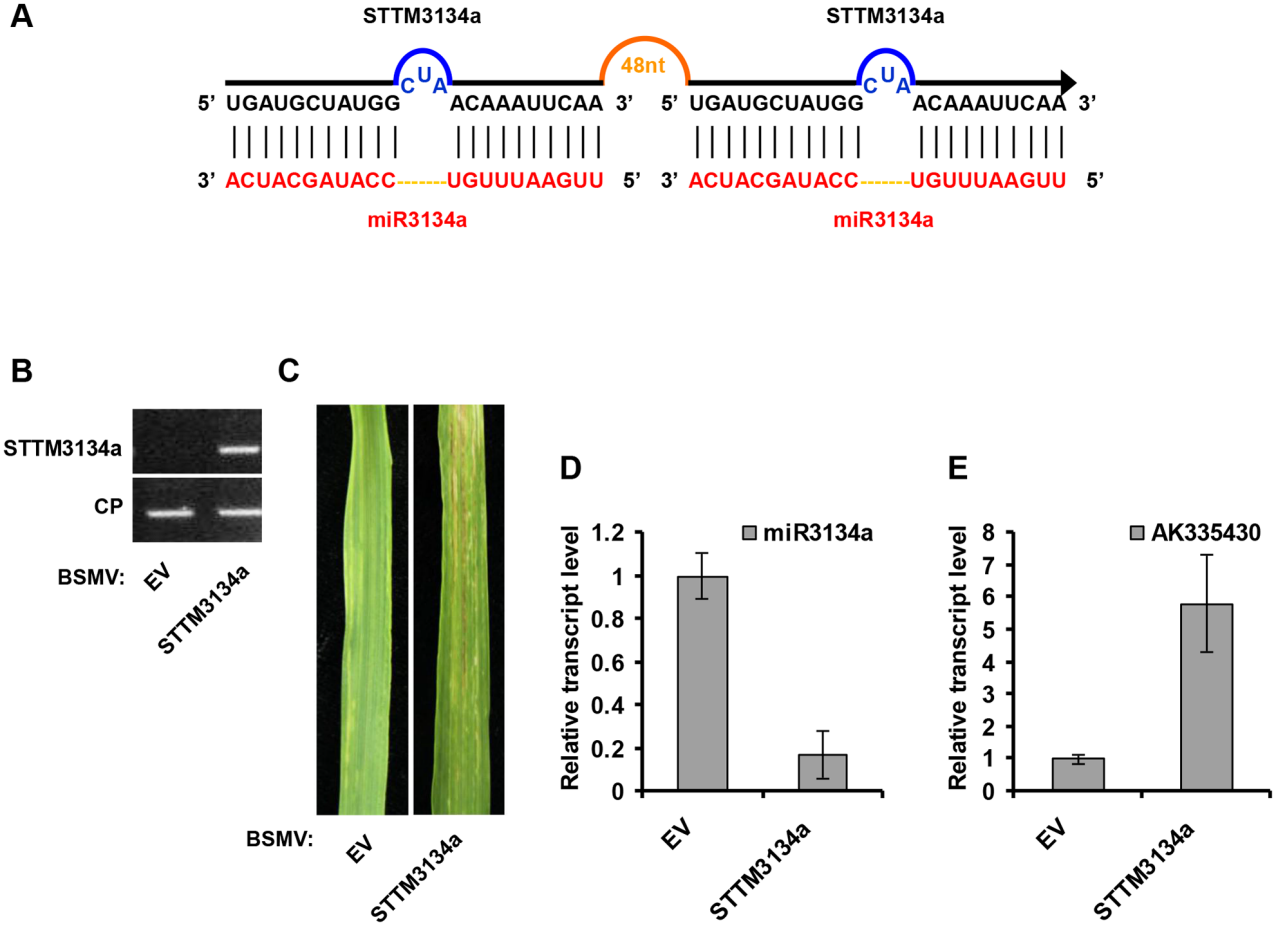
BSMV-based miR3134a silencing using the STTM approach in wheat. (**A**) Diagrammatic representation of STTM3134a structure. (**B**) Semiquantitative RT-PCR assays detection of STTM3134a structure expression in wheat infected with BSMV-EV and with BSMV-STTM3134a. CP, coat protein of BSMV. (**C**) The 4^th^ leaves of wheat infected with BSMV-EV (left) and with BSMV-STTM3134a (right) were photographed at 20 dpi. (**D**) Stem-loop RT-PCR together with real-time qPCR detection of mature miR3134a relative transcript level in wheat infected with BSMV-EV and with BSMV-STTM3134a. Error bars represented SE of three representing experiments from four replicates. (**E**) Real-time RT-PCR analysis of mRNA levels of miR3134a target AK335430 in BSMV-EV control and plants expressing STTM3134a structure. Error bars representing SE were calculated from three replicates.

**Fig 6:**
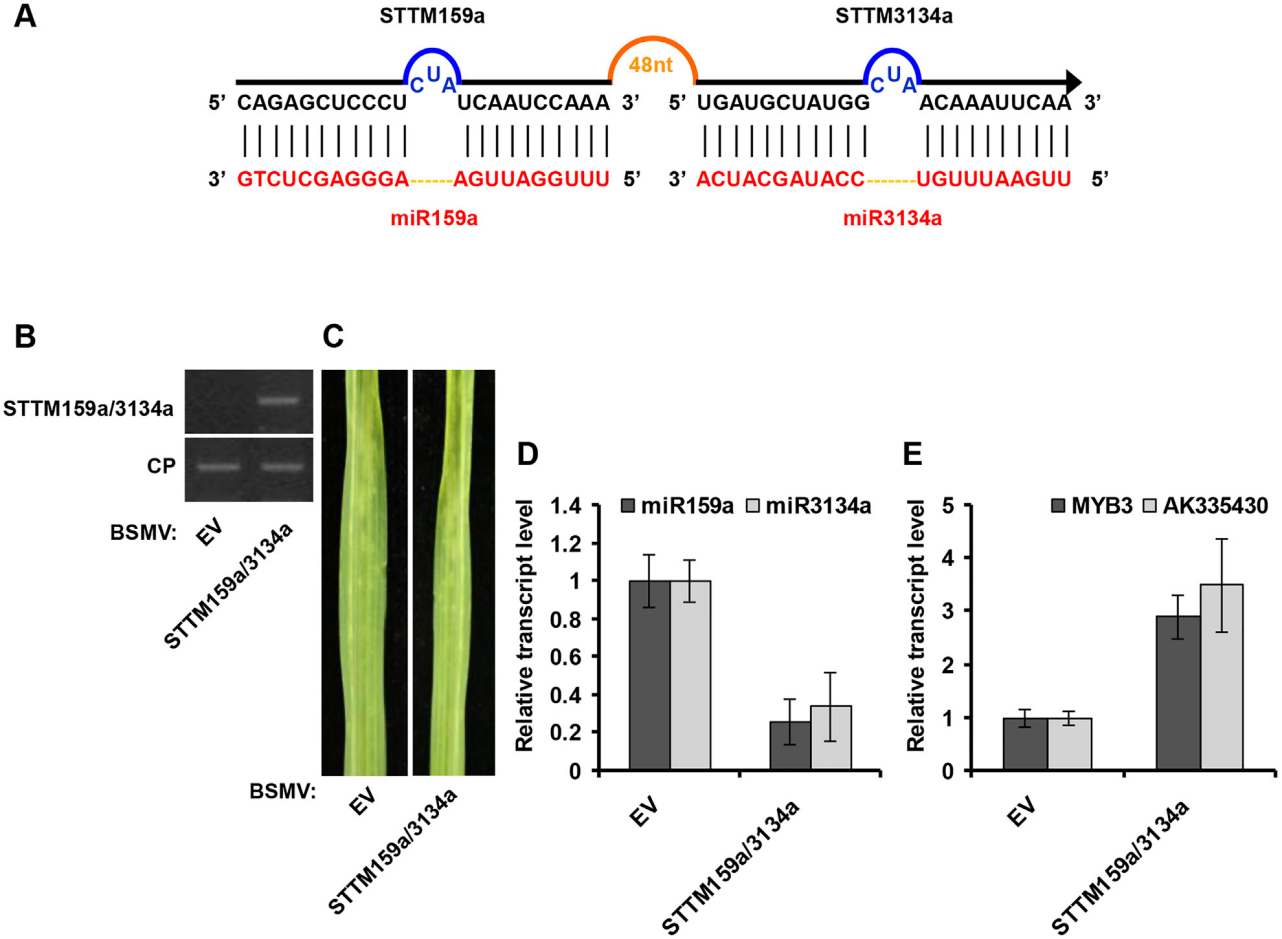
Simultaneous silencing of miR159a and miR3134a using the BSMV expressing STTM approach in wheat. (**A**) Diagrammatic representation of STTM159a/3134a structure. (**B**) Semiquantitative RT-PCR assays detection of STTM159a/3134a structure expression in wheat infected with BSMV-EV and with BSMV-STTM159a/3134a. CP, coat protein of BSMV. (**C**) The 4^th^ leaves of wheat infected with BSMV-EV (left) and with BSMV-STTM159a/3134a (right) were photographed at 20 dpi. (**D**) Stem-loop RT-PCR together with real-time qPCR detection of mature miR159a and miR3134a relative transcript level in wheat infected with BSMV-EV and with BSMV-STTM159a/3134a. Error bars represented SE of three representing experiments from four replicates. (**E**) Real-time RT-PCR analysis of mRNA levels of miR159a target *TaMYB3* and miR3134a target AK335430 in BSMV-EV control and plants expressing STTM159a/3134a structure. Error bars representing SE were calculated from three replicates.

We also tested whether BSMV can inhibit miRNA activity by BSMV-based STTM expression. For this purpose, we generated STTM against miR59a (STTM159a, for silencing of miR159a using STTM strategy) or against miR3134a (STTM3134a, for silencing of miR3134a using STTM strategy), and cloned it into pCaBS-γ-LIC vector to generate BSMV-STTM159a or BSMV-STTM3134a, respectively (Figs 4A and 5A). Similar to BSMV-MIM159a and BSMV-MIM3134a plants, obvious BSMV symptoms were visible in the upper noninoculated leaves when wheat plants were infected with BSMV carrying STTM sequences (Figs 4C and 5C). Accompanied with viral expression of STTM159a and STTM3134a (Figs 4B and 5B), mature miR159a and miR3134a level were lower (Figs 4D and 5D) while the mRNA level of *TaMYB3* and AK335430 was higher (Figs 4E and 5E) in STTM159a- and STTM3134a-expressing plants than in BSMV-EV infected plants. Furthermore, to simultaneously silence two endogenous miRNAs, we performed BSMV-based expression of STTM structure targeting miR159a/3134a (STTM159a/3134a, for simultaneous silencing of miR159a and miR3134a using STTM strategy; Fig 6A) in wheat plants. Semiquantitative RT-PCR confirmed that STTM159a/3134a sequence was expressed in BSMV-STTM159a/3134a inoculated plants (Fig 6B). BSMV symptoms were visible in the upper noninoculated leaves when wheat plants were infected with BSMV-STTM159a/3134a (Fig 6C). Moreover, the relative transcript level of mature miR159a and miR3134a were reduced in BSMV-STTM159a/3134a infected plants (Fig 6D), meanwhile the level of the *TaMYB3* and AK335430 mRNA were indeed increased (Fig 6E) in BSMV-STTM159a/3134a infected plants than in BSMV-EV infected plants. Taken together, our results suggest that BSMV-based miRNA silencing system using either MIM or STTM can effectively suppress miRNAs function, and this system can be applied to study the function of uncharacterized miRNAs in common wheat.

## Discussion

In this study, we demonstrated that the modified BSMV-mediated miRNA silencing system could be utilized to inhibit endogenous miRNAs activity in common wheat. Using this system, we successfully silenced miR159a and miR3134a in wheat. Indeed, the corresponding MIM or STTM sequences were detected and the mRNA levels of miR159a or miR3134a target gene increased in plants infected with BSMV expressing MIM or STTM sequences, but not in BSMV-EV infected plants. Our results indicated the BSMV-based miRNA silencing system by overexpressing miRNA target mimics was efficient in silencing endogenous miRNAs in wheat.

Hexaploid wheat, *Triticum aestivum* L. (2n = 6× = 42; genomes AABBDD) is one of the most staple crops in the world due to its high yield and nutritional and processing qualities. With the progress of draft genomes for bread wheat [33], it’s A-genome progenitor *Triticum urartu* (2n = 14; AA) [30] and its D-genome progenitor *Aegilops tauschii* (2n = 14; DD) [34], more and more wheat miRNAs have been annotated based on the whole-genome shotgun sequencing strategy. For a given miRNA locus in a diploid, there are three loci in a hexaploid, or more if the locus was duplicated in the diploid or tetraploid progenitor species or duplicated after the allopolyploid event, which might lead to a particularly large set of miRNAs at the genome scale [35]. Traditional methods to the interrogation of gene function rely on the generation and characterization of genetic mutants [36]. Such genetic approaches are not easily applicable to miRNAs due to their small size and the fact that many miRNA families are composed of multiple members with potentially overlapping functions. The ideal strategy to exploring the functions of miRNAs is the simultaneous blockage of several members of the miRNAs family so as to reveal the effects of derepressing all the target genes through a single genetic transformation event [16].

In plants, a minority of the annotated miRNA gene families are conserved between plant families, while the majority are family or species specific [37]. Many canonical miRNAs are conserved and some regulate conserved targets and display conserved functions among plant kingdom. The wheat-specific miRNAs are particularly interesting because they may function in a species-specific manner in wheat growth and development [35]. miR159 is conserved in monocot and dicot plants while miR3134 belongs to wheat- and barley-specific miRNAs family. miR159a is one of several miR159 family members with higher expression levels in wheat leaves. In contrast, the relative transcript level of mature miR3134a is much lower than miR159a in wheat leaves during different developmental stage (S1 Fig). Thus, miR159 and miR3134 are selected as tester miRNAs in this study.

Overexpression and silencing of miRNAs are two of the most widely used reverse-genetic strategy to study miRNA function [18,19]. Traditional approaches to study the functions of miRNAs usually need tedious and time-consuming work to generate the stable transgenic plants [16]. In contrast, the currently described BSMV-based miRNA silencing system possesses several advantages over other functional assays for plant miRNAs. First, BSMV-based miRNA silencing system is efficient and quick, and miRNA silencing mediated phenotypes will be observed within 4 weeks. Second, BSMV-based miRNA silencing system does not require complicated stable transformation procedure of wheat and only needs the simple agroinfitration technique for miRNA silencing. This is particularly useful for functional characterization of miRNAs whose knockout or knockdown might bring about sporophytic or gametophytic lethality in transgenic lines and for wheat crops that are not amenable to stable genetic transformation. Third, besides natural host, barley and wheat, BSMV has a wide host range, such as *Brachypodium distachyon*, *Zea mays*, *Oryza sativa* and *Avena sativa* [29]. BSMV-based miRNA silencing system can be applicable for miRNA functional analysis in these plants. Therefore, we introduce the modified BSMV vectors into wheat plants with a much simpler but effective technique. Our results demonstrate that BSMV-based miRNA silencing system can be used to evaluate the functions of endogenous miRNA genes. The modified BSMV vector may facilitate to high-throughput screen the targets of miRNAs and to characterize endogenous miRNA function in wheat crops. As above-mentioned, we employ this BSMV miRNA target mimics expression vector to elucidate the target genes of endogenous miR159a and miR3134a via transient down-regulation, which is convenient for us to determine target genes of endogenous miRNAs *in vivo*.

In summary, miRNA silencing approach using miRNA target mimic and STTM, together with the nature of the modified BSMV-based miRNA silencing system, have great potential for functional characterization of endogenous miRNAs in common wheat.

## Materials and Methods

### Plant materials

*N*. *benthamiana* plants are grown in a controlled environment at 25°C with a 14-h-light/ 10-h-darkness photoperiod. *Arabidopsis thaliana* plants (Columbia-0 background) are grown in long days (16-h-light/ 8-h-darkness) at 23°C. Wheat plants (YM158) used for BSMV-based miRNA silencing experiment are grown in pots in a green house with 16-h-light/ 8-h-darkness cycle until the two-leaf stage. After inoculated with BSMV, YM158 plants are transferred to a climate chamber at 23–25°C for the evaluation. For each biological replicates, six YM158 seeds are sown in one pot of 12 centimeter (cm) diameter, and 2 pots for per BSMV construct. Totally, 10–12 wheat plants of two-leaf stage are prepared for BSMV inoculation. Wheat materials are collected from three biological replicates.

### Vector constructions

*AtIPS1*-based target mimic against miR159a (MIM159a, for silencing of miR159a using *AtIPS1* sequence as backbone) is constructed as follows. *AtIPS1* sequence is obtained from *Arabidopsis* plants (see RNA and PCR Analysis section). Using the method previously reported, miR159a target mimic sequence (5’-CAGAGCTCCCTCTATCAATCCAAA-3’, Fig 2A) is constructed into *AtIPS1* backbone by overlap PCR to replace AthmiR399 target mimic sequence [14,15]. The primer groups of overlap PCR are J1/J4 and J2/J3. MIM159a is added LIC adaptors for linking with BSMV vector. *AtIPS1*-based target mimic against miR3134a (MIM3134a, for silencing of miR3134a using *AtIPS1* sequence as backbone) is constructed by the same strategy using overlap PCR primers J1/J6 and J2/J5. miR3134a target mimic sequence is 5’-TGATGCTATGGCTAACAAATTCAA-3’ (Fig 3A). Primers used in this study are listed in S1 Table.

STTM159a (for silencing of miR159a using STTM strategy) is constructed as follows. Primers with LIC adaptor, corresponding target mimic of miR159a, and STTM 48nt spacer (5’-GTTGTTGTTGTTATGGTCTAATTTAAATATGGTCTAAAGAAGAAGAAT-3’) are employed to PCR amplify STTM159a molecules using primers J9/J10 and J7/J8 (Fig 4A). STTM159a is added LIC adaptors for linking with BSMV vector. STTM3134a (for silencing of miR3134a using STTM strategy) and STTM159a/3134a (for simultaneous silencing of miR159a and miR3134a using STTM strategy) are constructed by the same method using primer groups J13/J14, J11/J12 and J9/J14, J7/J12, respectively (Fig 5A). Primers used in this study are listed in S1 Table.

The MIM or STTM fragments with LIC adaptors are cloned into BSMV-γb using the LIC protocol as described [29]. pCaBS-α, pCaBS-β, pCaBS-γ-LIC plamids for BSMV LIC protocol are reserved by our lab. First, MIM or STTM fragments with LIC adaptors reaction system: MIM or STTM fragments with LIC adaptors, about 100–200 ng; 100 mM dATP (Promega), 1.0 μL; 100×BSA (New England Biolabs), 0.1 μL; 10×buffer2 (New England Biolabs), 1.0 μL; T4 DNA polymerase (New England Biolabs), 0.2 μL; ddH_2_O up to 10 μL. All reagents mixture is placed at 22–25°C for 30 minutes, followed by 75°C for 30 minutes, and finally stored at 4°C. Second, pCaBS-γ-LIC reaction system: pCaBS-γ-LIC plasmid DNA digested by *ApaI* (New England Biolabs), about 20 ng; 100 mM dTTP (Promega), 1.0 μL; 100×BSA (New England Biolabs), 0.2 μL; 10×buffer2 (New England Biolabs), 2.0 μL; T4 DNA polymerase (New England Biolabs), 0.4 μL; ddH_2_O up to 20 μL. All reagents mixture is placed at 22–25°C for 30 minutes, followed by 75°C for 30 minutes, and finally stored at 4°C. Third, all the treated fragments and 2.0 μL of the treated pCaBS-γ-LIC vector are mixed. Mixture is placed at 66°C for 2 minutes and then cooled to room temperature. Then the final mixture can be transformed to *E*. *coli*. All constructs are confirmed by DNA sequencing.

### BSMV-based miRNA silencing experiment

BSMV-based miRNA silencing experiment is performed as described [29]. Constructs of pCaBS-α, pCaBS-β, pCaBS-γ-LIC derivatives (MIM159a, MIM3134a, STTM159a, STTM3134a and STTM159a/3134a) are transformed into *Agrobacterium* (*A*. *tumefaciens* strain EHA105). The *Agrobacterium* suspensions of OD_600_ = 0.8 are mixed at 1:1:1 ratio (pCaBS-α: pCaBS-β: each pCaBS-γ-LIC derivative) and infiltrated in *N*. *benthamiana* leaves. Agroinfiltrated *N*. *benthamiana* leaves can provide excellent sources of virus for secondary BSMV infections in wheat plants. The *N*. *benthamiana* sap is extracted from leaves with BSMV symptom at about 12 days post infiltration, ground in 20 mM Na-phosphate buffer (pH7.2) containing 1% celite, and the sap is mechanically inoculated onto the first two emerging leaves of wheat. Infected wheat plants are further grown for 14–21 d to allow emergence of new leaves displaying viral symptoms. Segments of the 4th leaves of BSMV-infected wheat plants are collected for detection from three biological replicates per construct.

### RNA and PCR analysis

Total RNAs are extracted from three independent biological replicates of *Arabidopsis* plants, BSMV-infected *N*. *benthamiana* leaves and BSMV-infected 4^th^ leaves of wheat with TRIzol reagent as described by the manufacturer (Invitrogen), and treated with Dnase I. About 2 mg of total RNA and M-MLV Reverse Transcriptase (Promega) are further used for reverse transcription. For coding genes reverse transcription, first-strand cDNA is synthesized using Oligo (dT)_18_. For miRNA reverse transcription, specifically designed end-point stem-loop reverse transcription primers are used, and follow the procedures described by Chen [38] and Varkonyi-Gasic [39]. Primers J21 and J23 are used for miR159a and miR3134a, respectively; primer J17 is used for *U6* (*U6* stands for *U6* spliceosomal RNA), and obtained cDNA is diluted 10 times and used for further analysis. Real-time RT-PCR assays with three technical replicates are performed using StepOne real-time system (Applied Biosystems) and GoTaq qPCR Master Mix (Promega, A6001). MiRNA forward primer J20 and J22 are respectively used with miRNA universal reverse primer J15 to quantify the relative transcript levels of mature miR159a and miR3134a. Real-time RT-PCR components for miRNA are as follows: 2×GoTaq qPCR Master Mix 5 μL, diluted cDNA 1 μL, miRNA forward primer 0.2 μL, miRNA universal reverse primer 0.2 μL, ddH_2_O up to 10 μL. Real-time RT-PCR conditions are as follows: 95°C for 5 min, followed by 35–40 cycles of 95°C for 5 s, 60°C for 10 s, and 72°C for 1 s. For melting curve analysis, denature samples at 95°C, then cool to 65°C at 20°C per second [39]. For the determination of target genes *MYB3* and AK335430, gene-specific primer pairs (J28/J29 and J30/J31) spanning the miRNA-guided cleavage site are used. Primer pairs J16/J17 and J18/J19 are used for the detection of *U6* and *Actin* which served as internal reference gene for miRNAs and protein-coding genes, respectively. Error bars representing standard error (SE) are calculated from three biological replicates per construct. *AtIPS1* backbone sequence is amplified by PCR primers J26 and J27. Semiquantitative RT-PCR assays [40] with three technical replicates using *N*. *benthamiana* cDNA as template, are performed for amplification of MIM159a, MIM3134a, STTM159a, STTM3134a, STTM159a/3134a and BSMV coat protein (CP) sequences; primer pairs J26/J27 and J24/J25 are used. The semiquantitative RT-PCR products are analyzed by electrophoresis on a 1% agarose gel in 1× Tris-acetate EDTA (TAE) buffer. All primers are listed in S1 Table.

Sequence data for genes used in this article can be found under GenBank accession numbers KC775781 (*TaActin*), X63066 (*TaU6*), AY615200 (*TaMYB3*), AK335430 (target gene of miR3134a), NM_180219 (*AtIPS1*) and U35772 (BSMV coat protein).

## Supporting Information

S1 Fig Relative transcript level of mature miR159a and miR3134a in wheat leaves during different developmental stages. (DOC)

S1 Table Primers used in vector construction and PCR analysis. (DOC)
