# Supplementary material for: Barley Stripe Mosaic Virus (BSMV) Induced MicroRNA Silencing in Common Wheat (Triticum aestivum L.)
Source: PLoS One. 2015 May 8;10(5):e0126621. doi: 10.1371/journal.pone.0126621 (PMC4425524; doi:10.1371/journal.pone.0126621)
Supplement: S1 Fig — (DOC) [file pone.0126621.s001.doc]

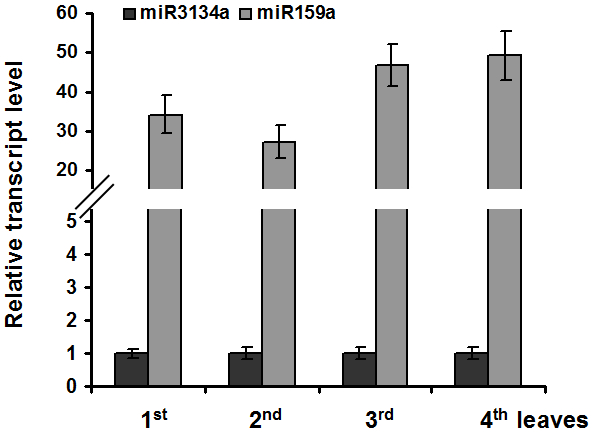


**S1 Fig**. **Relative transcript level of mature miR159a and miR3134a in wheat leaves during different developmental stages.** 1^st^, 2^nd^, 3^rd^, and 4^th^ leaves were collected from four-leaf stage wheat plants, respectively. For each biological replicate, at least three 1^st^ leaves, three 2^nd^ ones, three 3^rd^ ones, and three 4^th^ ones were detached, respectively. Stem-loop RT-PCR together with real-time quantitative PCR (qPCR) assays were performed for detection of mature miR159a and miR3134a relative transcript level in different leaves. Error bars represented standard error (SE) of three representing experiments from four replicates.
