# Supplementary material for: Barley Stripe Mosaic Virus (BSMV) Induced MicroRNA Silencing in Common Wheat (Triticum aestivum L.)
Source: PLoS One. 2015 May 8;10(5):e0126621. doi: 10.1371/journal.pone.0126621 (PMC4425524; doi:10.1371/journal.pone.0126621)
Supplement: S1 Table — (DOC) [file pone.0126621.s002.doc]

| **Number** | **Primers** | **Primer sequence (5’-3’)** |
| --- | --- | --- |
| J1 | IPS1-adpA ^a^ | AAGGAAGTTTAACACACAAAGAACACACAACG |
| J2 | IPS1-adpB ^a^ | AACCACCACCACCGTAAGAAAAATGGCCATCCCCTAGC |
| J3 | IPS1-159mim-F ^b^ | CGAGGGGAACCGAAGCTTTTGGATTGATAGAGGGAGCTCTG |
| J4 | IPS1-159mim-R ^b^ | TTATCTCCCTCTAGAAACAGAGCTCCCTCTATCAATCCAAA |
| J5 | IPS1-3134mim-F ^b^ | CGAGGGGAACCGAAGCTTTGAATTTGTTAGCCATAGCATCA |
| J6 | IPS1-3134mim-R ^b^ | TTATCTCCCTCTAGAAATGATGCTATGGCTAACAAATTCAA |
| J7 | STTM-159-adpA ^a^ | AAGGAAGTTTAACAGAGCTCCCTCTATCAATC |
| J8 | STTM-159-adpB ^a^ | AACCACCACCACCGTTTTGGATTGATAGAGGGAG |
| J9 | STTM-159-F ^C^ | CAGAGCTCCCTCTATCAATCCAAAGTTGTTGTTGTTATGGTCTAATTTAAATATGGTC |
| J10 | STTM-159-R ^C^ | TTTGGATTGATAGAGGGAGCTCTGATTCTTCTTCTTTAGACCATATTTAAATTAGACC |
| J11 | STTM-3134-adpA ^a^ | AAGGAAGTTTAATGATGCTATGGCTAACAAAT |
| J12 | STTM-3134-adpB ^a^ | AACCACCACCACCGTTTGAATTTGTTAGCCATAG |
| J13 | STTM-3134-F ^C^ | TGATGCTATGGCTAACAAATTCAAGTTGTTGTTGTTATGGTCTAATTTAAATATGGTC |
| J14 | STTM-3134-R ^C^ | TTGAATTTGTTAGCCATAGCATCAATTCTTCTTCTTTAGACCATATTTAAATTAGACC |
| J15 | SL-R | GTGCAGGGTCCGAGGT |
| J16 | U6-F | GGGGACATCCGATAAAATTGG |
| J17 | U6-R | GGACCATTTCTCGATTTGTGC |
| J18 | Actin-F | TGGCACCCGAGGAGCACC |
| J19 | Actin-R | GTAACCTCTCTCGGTGAG |
| J20 | miR159-F ^d^ | CGGCGTTTGGATTGAAGGGA |
| J21 | miR159-SL ^e^ | GTCGTATCCAGTGCAGGGTCCGAGGTATTCGCACTGGATACGACCAGAGC |
| J22 | miR3134-F ^d^ | GCCGCTTGAATTTGTCCATA |
| J23 | miR3134-SL ^e^ | GTCGTATCCAGTGCAGGGTCCGAGGTATTCGCACTGGATACGACTGATGC |
| J24 | BSMV-CP-F | ATGCCGAACGTTTCTTTGAC |
| J25 | BSMV-CP-R | TCACGCTTCCTCGGCATCTG |
| J26 | IPS1-F | CACACAAAGAACACACAACG |
| J27 | IPS1-R | AAGAAAAATGGCCATCCCCTAGC |
| J28 | MYB3-RT-F | TAAATGGCACCTTCTCTACTTC |
| J29 | MYB3-RT-R | TGTCGGAGACTGAAGGTAGG |
| J30 | 335430-RT-F | GAGTGGCACGAACTGGAGGA |
| J31 | 335430-RT-R | TTACCGCTCTGACCACCATC |

**S1 Table. Primers used in vector construction and PCR analysis.** ^a^, Adaptor used for linkage of BSMV vector and *AtIPS1* backbone or STTM; ^b^, Target mimic sequence is underlined; ^c^, STTM mimic sequence is underlined; ^d^, MiRNA sequence is underlined; ^e^, Reverse-complement sequence of miRNA is underlined.
